# 2024 Acknowledgment of *mBio* Reviewers

**DOI:** 10.1128/mbio.03938-24

**Published:** 2025-02-05

**Authors:** Arturo Casadevall

**Affiliations:** 1 Johns Hopkins Bloomberg School of Public Health, Baltimore, Maryland, USA

## EDITORIAL

On behalf of the editors of *mBio*, I gratefully acknowledge the following members of our Early-Career Editorial Board who served as reviewers for the journal in 2024. These dedicated and well-qualified early-career researchers are a welcome addition to our community of reviewers.

Rhea Abisado-Duque

Rachy Abraham

Philip Adams

Jontana Allkja

C. J. Anderson

Frederik Bak

Maksym Bobrovskyy

Ian Boys

Joshua Burns

Gustavo Caballero-Flores

Pengbo Cao

Aitor Casas-Sanchez

Yi-Wei Chen

Prashant Damke

Marcos de Moraes

Heather Evans

Anat Florentin

Muinah Fowora

Katsuya Fuchino

Kristie Goughenour

Wenyu Gu

Caitlyn Holmes

Jun Huang

Yolanda Huang

Zahra Islam

Marta Jambrina

Jose Jimenez-Guardeño

Sachie Kanatani

Dorota Kmiec

Dilip Kumar

Bradley Laflamme

Yong Lai

Noelia Lander

Apostolos Liakopoulos

Richard Lindsay

Gideon Mamou

Sinalo Mani

Katrina Mar

Ameya Mashruwala

Benjamin Morehouse

Budhaditya Mukherjee

Nhu Nguyen

Coral Pardo-Esté

Grace Pold

Aimee Potter

Shannon Righi

Juan Rivera-Correa

Jesús Romo

Severin Ronneau

Brittany Ross

Pierre Santucci

Zeyang Shen

Iart Shytaj

Chandni Sidhu

Brady Spencer

Benjamin Stein

Madison Stellfox

Micaela Tosi

Chih-Hsuan Tsai

Aayushi Uberoi

Lucas Ustick

Norman van Rhijn

José Vargas-Muñiz

Laura Veschetti

Xinru Wang

Yen-Wen Wang

Zhenting Xiang

Merve Zeden

Xuhui Zheng

On behalf of the editors of *mBio*, I gratefully acknowledge the following individuals, who served as reviewers for the journal from November 2023 to November 2024. Their time and effort in reviewing articles have been essential in ensuring the high quality of our publications, and their help is greatly appreciated.

Jeffrey Abbott

Abdirahman Abdi

Rhea Garces Abisado-Duque

Rachy Abraham

Robert Abramovitch

Brian Ackley

Philip Adams

Cynthia Ayefoumi Adinortey

Herve Agaisse

Vinayak Agarwal

Surya Aggarwal

Hector Aguilar

Jesus Aguirre

Tero Ahola

Umesh Ahuja

Xingbin Ai

Christopher Aiken

Vishukumar Aimanianda

Patrícia Albuquerque

Borja Aldeguer-Riquelme

Maria-Luisa Alegre

Alí Alejo

Gladys Alexandre

Juan Alfonzo

Holly Scott Algood

Youssif Ali

Kylie Allen

Lee-Ann Allen

Kyle R. Allison

Jontana Allkja

Luke P. Allsopp

Fadi G. Alnaji

Francis Alonzo

Craig Altier

Shoshy Altuvia

Diego Alvarez

Fairoz Ali Al-Wrafy

Pedro M. Alzari

Zandrea Ambrose

Amal Amer

Jorge Amich

Mary Cloud Ammons Anderson

Christopher Anderson

Deborah M. Anderson

Matthew Zack Anderson

Sarah Anderson

Dan Andersson

David Andes

Sarah Andrews

Elliot Androphy

Anastasia Antoniadou

Daniel Aridgides

Robert Arkowitz

Chelsie Elizabeth Armbruster

Jean Armengaud

Gustavo Arrizabalaga

Eric Arts

Divya Arunachalam

Sassan Asgari

Usama Ashraf

John M. Atack

Walter Atwood

Jacques Augenstreich

Noam Auslander

Ruben Avendaño-Herrera

Roi Avraham

Taj Azarian

Sheyda Azimi

Juhi Bagaitkar

Zeynep Baharoglu

Guangchun Bai

Joel Baines

Goran Bajic

Frederik Bak

Lauren Bakaletz

David Baker

Jonathon Baker

Mitchell Balish

Anand Ballal

Elizabeth Ballou

David Baltrus

Samiran Banerjee

Gert Bange

James Bangs

Lori Delorme Banks

Yiqiao Bao

Jeri D. Barak

Matthew F. Barber

Joseph T. Barbieri

Daniela Barilla

Bridget M. Barker

Soumik Barman

Riccardo Baroncelli

Jeremy J. Barr

John Barr

Frédéric Barras

Luther A. Bartelt

Ralf Bartenschlager

Jonelle T. R. Basso

Himanshu Batra

Buzz Baum

Andreas Bäumler

Victoria K. Baxter

Ethel Bayer-Santos

Özgür Bayram

Clifford J. Beall

Pascale B. Beauregard

David H. Bechhofer

Stephen Beckett

Stéphanie Bedhomme

Morgan Beeby

Christine Beemelmanns

Peter T. Beernink

Isabelle Bekeredjian-Ding

Joel G. Belasco

Jennifer Bell

Samantha L. Bell

Deborah Bell-Pedersen

George A. Belov

Jessica A. Belser

Graham John Belsham

Tom Beneke

Jose Bengoechea

Julien Bergeron

Marta Bermejo-Jambrina

Luiz E. Bermudez

Antonio Bertoletti

Sébastien Besteiro

Gilberto Betancor

Aadra Prashant Bhatt

Tapan Bhattacharyya

Yuhan Bi

Kay Bidle

Scott B. Biering

Emanuele G. Biondi

Rajesh Kumar Biswas

Wilbert Bitter

Leonora S. Bittleston

Gregory Bix

Nicolas Blanchard

Matthew Blango

Robert Blankenship

Jon S. Blevins

Catherine A. Blish

James B. Bliska

Carlos J. Blondel

Louis-Marie Bobay

Maksym Bobrovskyy

Ana Maria Bocsanczy

Helge Bode

Karl W. Boehme

Luis M. Bolaños

Michael Bölker

Joseph Boll

Jani Bolla

Paul L. Bollyky

Diane L. Bolton

Morgane Bomsel

Matteo Bonazzi

Joseph Bondy-Denomy

Paola Bonfante

Clotilde Bongrand

Kevin Bonham

Elizabeth Boon

Batbileg Bor

Seth Bordenstein

Juliano Bordignon

Thomas Boren

Joshua Borin

Katherine A. Borkovich

Sally Bornbusch

Alexander Borodavka

Juan Borrero

Angela M. Bosco-Lauth

Alberto Bosque

Jamie Botsch

Jason W. Botten

Evzen Boura

Stylianos Bournazos

Robert Bowers

Eric Boyd

Ethna Fidelma Boyd

Jeff M. Boyd

Ian N Boys

Zbynek Bozdech

Günter Brader

Peter J. Bradley

Marc Bramkamp

Kalvis Brangulis

Benjamin Bratton

Miriam Braunstein

Gerhard Braus

Andrew Bridges

Melinda Ann Brindley

Shaun Brinsmade

Shaun R. Brinsmade

Christopher C. Broder

Nichole A. Broderick

Lone Brøndsted

Mark Brönstrup

Christopher B. Brooke

Sam Brown

Linda Brubaker

Harald Bruessow

John H. Brumell

Sascha Brunke

Mark P. Brynildsen

Nicole R. Buan

Juliane Bubeck Wardenburg

Shilpa Buch

Iain Buchan

Nienke Buddelmeijer

Lindsay Burcham

Tessa Burch-Smith

Paul-Christian Burda

Joshua Burns

Nick Burton

Stephen Busby

Alessia Buscaino

Corine Buser

Bettina A. Buttaro

David J. Bzik

Gustavo Caballero Flores

Filipe Cabreiro

Elisabetta Cacace

Sarah Caddy

Ken Cadwell

Lílian Caesar

Melissa J. Caimano

Gabriella Campadelli-Fiume

Elizabeth Campbell

Joseph Campbell

Samuel K. Campos

David Cánovas

Tineke Cantaert

Rafael M. Cantón

Bin Cao

Pengbo Cao

Yi Cao

Yongchang Cao

Yuansheng Cao

Daiana Capdevila

Eric Caragata

Jan E. Carette

Allison F. Carey

Lars-Anders Carlson

Jane M. Carlton

Jason A. Carlyon

Stephen M. Carpenter

Jillian Maree Carr

John P. Carr

Mark Carrington

Agostinho Carvalho

Aitor Casas-Sanchez

James E. Cassat

Roberto Cattaneo

Simon Cauchemez

Cristobal Chaidez

Amitabh Chak

Romy Chakraborty

Patricia A. Champion

John Chan

Josephine R. Chandler

Christina C. Chang

Vincent Charron-Lamoureux

Som S. Chatterjee

Saurabh Chattopadhyay

Soraya Chaturongakul

Vishnu Chaturvedi

Methee Chayakulkeeree

Nanying Che

Haoyan Chen

Benjamin K. Chen

Chen Chen

Dima Chen

Jiandong Chen

Wenqing Chen

Xiao-Lin Chen

Yi-Wei Chen

Zhiwei Chen

Zhongzhou Chen

Ziqiang Cheng

Edith Chepkorir

Daniel S. Chertow

Frederic D. Chevalier

Fabienne F. V. Chevance

Claire Chewapreecha

Yueh-Hsiu Chien

Obinna Chijioke

Chetan E. Chitnis

Petr Chlanda

Moloko Cholo

Rimi Chowdhury

Graham Christie

Ewa Chrostek

Nohemi Cigarroa Toledo

Andrea Cimarelli

Ousmane H. Cissé

Cornelius J. Clancy

Catherine Clarke

Jonathan B. Clayton

Carolina Coelho

Rodrigo Cogni

James F. Colbert

Mattias Collin

Fabian Moritz Commichau

Alex A. Compton

Gabriela Nérida Condezo

Brian Conlon

Sarah A. Connolly

Nicholas K. Conrad

Isabelle Coppens

Brendan Cormack

Valerie Cortez

Peggy Cotter

James Cotton

Timothy L. Cover

Jeffery S. Cox

Rory Craig

Robert Cramer

Sebastien Crepin

Steven Gaines Cresawn

Sean Crosson

Laszlo N. Csonka

Valeria C. Culotta

Christina A. Cuomo

Marina Cvetkovska

Miroslaw Cygler

Michael Cynamon

Charles J. Czuprynski

Nsa Dada

Jan-Ulrik Dahl

Helong Dai

Colin Dale

Ankur B. Dalia

Julian Damashek

Prashant Purushottam Damke

Andrew Dancis

Ajai A. Dandekar

Gautam Dantas

Pranav Danthi

Sophie E. Darch

Sudip Das

Matthew Davey

Alan R. Davidson

Bryan W. Davies

J. Muse Davis

Simon Davy

Suzanne Dawid

Ellen Decaestecker

Kirk W. Deitsch

Champion Deivanayagam

Nicholas R. De Lay

Laura Ruth Delgui

Sarah Dellière

Maurizio Del Poeta

Neal A. Deluca

Antonella De Matteis

Jill A. Dembowski

Xin Deng

Yi Zhen Deng

Zengqin Deng

Nicole J. De Nisco

Jonathan J. Dennis

Keith M. Derbyshire

Mark Derbyshire

Isabelle Derré

Sanjay A. Desai

Albert Descoteaux

Johannes Dessens

Corrella S. Detweiler

Damien Devos

Priyankar Dey

Eric Déziel

Vijaykrishna Dhanasekaeran

Sayera Dhaubhadel

Eduardo Diaz

Thomas Dick

Robert P. Dickson

Sean A. Diehl

Andreas Diepold

Heidi Dierssen

Lars E. P. Dietrich

Stephen P. Diggle

Stephen DiGiuseppe

Jimmy D. Dikeakos

Joseph P. Dillard

Tatiana Dimitriu

Chan Ding

Chen Ding

Qiang Ding

Siyuan Ding

Victor J. DiRita

Dirk P. Dittmer

Linda K. Dixon

Armin Djamei

Julianne T. Djordjevic

Thomas Dobner

Rebecca Dodd

Tobias Doerr

Stephen K. Dolan

Maria Rosa Domingo-Sananes

Claudio Donati

Rebecca K. Donegan

Min Dong

Tao Dong

Xintong Dong

Allie Donlan

Sarah M. Doore

Pedro Dorado-Morales

Sarah E. F. D'Orazio

Daolong Dou

Zhicheng Dou

Benoît Doublet

Jordan Douglas

Badreddine Douzi

Elizabeth Draganova

Shaynoor Dramsi

Marlene Dreux

Djamel Drider

Arnold J. M. Driessen

Rebecca A. Drummond

David Dubnau

Rebecca M. Dubois

Katherine Duchesneau

Edward G. Dudley

Breck A. Duerkop

Iain G. Duggin

Katherine R. Duncan

Manoj T. Duraisingh

Jeffrey D. Dvorin

Jonathan Dworkin

Michelle Dziejman

Alison J. Eastman

Gregory D. Ebel

Taylor Eddens

Andrew Edwards

Marcus J. Edwards

Patrick Eichenberger

Iris Eisermann

Melissa Ellermann

Craig D. Ellermeier

Marie A. Elliot

Courtney K. Ellison

Bert Ely

Carolyn Elya

Mohamed Ezzat El Zowalaty

Philipp Engel

Pamela Engelberts

Susanne Engelmann

Luis Enjuanes

John Erb Downward

Joel Ernst

Jorge C. Escalante-Semerena

José A. Escudero

Aria Eshraghi

Evan A. Eskew

Manuel Espinosa-Urgel

Ronald Drew Etheridge

Heather M. Evans

Sarah Elisabeth Ewald

Franziska Faber

Oliver T. Fackler

Delisa Fairweather

Ahmad Fakhoury

Huizhou Fan

Jiangbo Fan

Xiaoqing Fan

Rubaiyea Farrukee

Carmen Faso

Ariberto Fassati

Denis Faure

Mathias Faure

Heather Feaga

Michael J. Federle

Anthony R. Fehr

Na Fei

Rena Feinman

Mario F. Feldman

Carl Feng

Jia-Xun Feng

Pinghui Feng

Yingang Feng

James Ferguson

Mariola J. Ferraro

Tiago R. Ferreira

Igor Fesenko

Paul D. Fey

David A. Fidock

Aretha Fiebig

Kenneth A. Fields

Joshua Fierer

Sergio R. Filipe

Sabine Fillinger

Alain Filloux

Peter Charles Fineran

Steven Finkel

Anthony John Fischer

Joseph Flaherty

Klas Flardh

Hansel M. Fletcher

Anat Florentin

Laura V. Florez

Thierry Fontaine

Suan-Sin Foo

Anja Forche

Kyle Ford

Adriana Forero

Diego Forni

Kevin Forsberg

Timothy J. Foster

Toshana L. Foster

Vance G. Fowler

Muinah Adenike Fowora

Julie M. Fox

Michael Fox

Ashwanth Francis

Andrew Frando

Carl Johan Franzén

Eric O. Freed

Dorte Frees

Eva Frickel

Michael Friedlander

Matthew B. Frieman

Bettina C. Fries

Timothy Friesen

Friedrich Frischknecht

Kathrin Sophie Fröhlich

Stefanie Fruhwürth

Julia Frunzke

Mayuree Fuangthong

Katsuya Fuchino

Coralie Fumeaux

Dana H. Gabuzda

Michaela Ulrike Gack

Marta M. Gaglia

Sara Gago

Liraz Galia

Tom Gallagher

Jean-Pierre Gangneux

Beile Gao

Feng Gao

Yuanjun Gao

Esther Garces

Brandon Garcia

Celia Garcia

Sarahi L. Garcia

Sarela Garcia-Santamarina

Ethan C. Garner

James Garnett

Robert F. Garry

Yves Gaudin

Susanne Gebhard

Patricia Geesink

Angie Gelli

Donovan German

Saeid Ghavami

Sean M. Gibbons

Julie Gibbs

David Giedroc

Danna Gifford

Tim Gilberger

Brad Paul Gilbertson

Teresa Gil-Gil

Stacey D. Gilk

Jason J. Gill

Michael S. Gilmore

Peter R Girguis

Mathieu Gissot

Philippe Glaser

Michael Glickman

Erin Gloag

Emile Gluck-Thaler

Camille Goemans

Shana K. Goffredi

Daniel Goldberg

Ido Golding

Piotr Golec

Lang Gong

Carlos F. Gonzalez

Lisandro Gonzalez

Irene González-Domínguez

Michael Good

Alan G. Goodman

Andrew Goodman

Joel M. Goodman

Heidi Goodrich-Blair

Felicia Goodrum

Rajaraman Gopalakrishnan

Susan Gottesman

Kristie Goughenour

Kathleen L. Gould

Celia W. Goulding

Campbell W. Gourlay

Alexandre Gouzy

Chhabi Kumar Govind

Grzegorz Jan Grabe

Marcin Grabowicz

Jeffrey A. Gralnick

Lone Gram

Michael D. Grant

André Alex Grassmann

Urs F. Greber

Hyatt C. Green

Alexander L. Greninger

Mark S. Gresnigt

Elizabeth A. Grice

Melinda R. Grosser

Anne Grove

Fabio Gsaller

Wenyu Gu

Xueli Guan

Pablo Guardado-Calvo

Marc-Jan Gubbels

Nancy Guillen

Allan J. Guimaraes

Pascale Guiton

Vadim M. Gumerov

John S. Gunn

Robert P. Gunsalus

Haitao Guo

Hui-Shan Guo

Ju-Tao Guo

Asiya Gusa

Jenna Guthmiller

Hubertus Haas

Ted Hackstadt

Maria Hadjifrangiskou

Daniel H. Haft

Martin W. Hahn

Matthias Hahn

Rong Hai

George Hajishengallis

Anders P. Hakansson

Sven Halbedel

James P. J. Hall

Rebecca A. Hall

Ruth M. Hall

Mehrad Hamidian

Marie-Louise Hammarskjold

Sven Hammerschmidt

Shichong Han

Andreas Handel

Blake M. Hanson

Bing Hao

Jianjun Hao

Weilong Hao

William R. Harcombe

Matthew J. Harke

Christopher Harmer

Eliza Harris

Steven Harris

Tajie Harris

Joe Harrison

Kevin S. Harrod

Duncan James Hart

Dennis J. Hartigan-O'Connor

Elizabeth L. Harvey

Amir Hassan

Karl A. Hassan

Asma Hatoum-Aslan

Vasili Hauryliuk

Gerald Hazelbauer

Tracy H. Hazen

Cynthia Y. He

Hongbin He

Aoife T. Heaslip

Nicholas S. Heaton

Tobias E. Hector

Brian P. Hedlund

P. Scott Hefty

Johann Heider

Christine Heilmann

Kai Heimel

Ekaterina E. Heldwein

John Helmann

Jeffrey P. Henderson

David R. Hendrixson

Tania Henriquez

Jennifer K. Herman

Bruno Hernáez

Elena Herrera Carrillo

Alfredo H. Herrera-Estrella

Martina Herrmann

Anat A. Herskovits

Mark C. Herzberg

Seth B. Herzon

Wolfgang Hess

Dagmar Heuer

Lena J. Heung

Paul G. Higgins

Penelope Higgs

Hubert Hilbi

Peter Hill

B. Joseph Hinnebusch

Mengfei Ho

Craig A. Hodges

Martin Hoenigl

Katharina Höfer

Ron Hoham

Caitlyn L. Holmes

Jie Hong

Saul M. Honigberg

Grace Hooks

David C. Hooper

Yasuhiko Horiguchi

Alexander Horswill

Benjamin A. Horwitz

Michael Hothorn

Ya-Ming Hou

Yiping Hou

Edith N. G. Houben

Jon Houseley

Peter M. Howley

Andrew J. Hryckowian

Boli Hu

Jianming Hu

Yilin Hu

Juan Huang

Jun Huang

Kuo-Yang Huang

Lu Huang

Yao-Wei Huang

Yolanda Huang

Luciano F. Huergo

Phil Hugenholtz

Christina M. Hull

Judd F. Hultquist

Katherine R. Hummels

Ian R. Humphreys

Christopher Hunter

Ryan C. Hunter

Julian G. Hurdle

Tracy Hussell

Geelsu Hwang

Kevin Hybiske

Joseph M. Hyser

Giuseppe Ianiri

Michael Ibba

Carolyn Ibberson

Hiroaki Ihara

Saheed Imam

James Imlay

Jean-Luc Imler

Barbara Imperiali

Thomas J. Inzana

Ronald M. Iorio

Javier Irazoqui

Ralph R. Isberg

Zahra Fatima Islam

Masahiro Ito

Paniz Izadi

Ray William Izquierdo Lara

Nuria Izquierdo-Useros

Conrad Izydorczyk

Marcelo Jacobs-Lorena

Alexander L. Jaffe

Swati Jain

Anne Jamet

Eric Jan

Guilhem Janbon

Andrea Jani

Fabrice Jean-Pierre

Lars Jelsbak

Urs Jenal

Kirk D. C. Jensen

Ki Jun Jeong

Ann Jerse

Honglin Jia

Cong Jiang

Guochun Jiang

Ping Jiang

Yuji Jiang

Diego Javier Jimenez Avella

Jose M. Jimenez-Guardeño

Fernanda Jiménez Otero

Jörgen Johansson

Evan John

Clare Jolly

Kristina Jonas

Clinton J. Jones

R. Brad Jones

Matthew A. Jorgenson

Joyce Jose

Eric Josephs

Quentin Josso

Thomas Jové

Michael J. Joyner

Feng Ju

Katy Juárez

Howard S. Judelson

Nathalie Juge

Heinrich Jung

Jae U. Jung

Kirsten Jung

Kwang-Woo Jung

Yue Junming

Praveen Rao Juvvadi

David Kadosh

Joerg Kaemper

Barbara C. Kahl

Kenji Kai

Manjula Kalia

Marina G. Kalyuzhnaya

Shaden Kamhawi

Jeremy Phillip Kamil

Jen Kane

Melissa Kane

Hyun Ah Kang

Cheng-Yuan Kao

Ulrike Kappler

Shraddha Karanth

Talia L. Karasov

Shahid Karim

Rajendra Karki

Lisa Karstens

Justin R. Kaspar

Takao Kasuga

Rachel A. Katzenellenbogen

Andrew Kau

Alexis Kaushansky

Barbara I. Kazmierczak

Daniel B. Kearns

Lance Edward Keller

David Kennedy

Linda J. Kenney

Robyn S. Kent

Lee J. Kerkhof

Nadeem Khan

Denys A. Khaperskyy

Anupama Khare

Megan R. Kiedrowski

Arlinet Kierbel

Patricia J. Kiley

Hwan Keun Kim

Kami Kim

Peter E. Kima

Matthew Kimber

Samantha Jane King

Taroh Kinoshita

Anja Kipar

James E. Kirby

Natalia V. Kirienko

Theo N. Kirkland

P. J. Klasse

Eric A. Klein

Uli Klümper

Dorota Kmiec

David M. Knipe

Katrin Knittel

Leigh Knodler

Eric Koch

Joshua Kochanowsky

Alain Kohl

Steffen Kolb

Jay Kolls

Heidi H. Kong

Ning Kong

James B. Konopka

Asja Korajkic

Konstantinos Aristomenis Kormas

Anita A. Koshy

Yanjun Kou

Vassili N. Kouvelis

Ákos T. Kovács

Orkide O. Koyuncu

Lukasz Kozubowski

Michael Kracht

Peter Kraiczy

Florian Krammer

Bryan Krantz

Ksenia Krasileva

Robert Krause

Tino Krell

Susanne Kreuzer-Redmer

Revathy Krishnamurthi

Bernhard Krismer

Laurie T. Krug

Mart Krupovic

Damian J Krysan

Anna Kuchina

Ulrich Kück

Jens H. Kuhn

Thijs Kuiken

Ritwij Kulkarni

Smita Kulkarni

Ayush Kumar

Dilip Kumar

Mukesh Kumar

Sanjai Kumar

Rolf Kümmerli

Caroline Kumsta

Biki Kundu

Oliver Kurzai

Tomomi Kuwahara

Elena Kuzmin

Mamuka Kvaratskhelia

Peter D. Kwong

Borden Lacy

Bradley John Laflamme

Olga Maria Lage

Leo Lahti

Yong Lai

Erh-Min Lai

Laimonis A. Laimins

Seema Lakdawala

Tommy T. Y. Lam

Marco Pio La Manna

Rebecca L. Lamason

Mart M. Lamers

Guoyu Lan

Lefu Lan

Nathaniel Landau

Nathaniel Roy Landau

Noelia Lander

Ryan A. Langlois

Gordon Langsley

Jason Larabee

Joe Larkin

Christopher Larock

Luis Larrondo

Gerald Larrouy-Maumus

Inigo Lasa

Cornelia Lass-Floerl

Adam S. Lauring

Martin Frederik Laursen

Catherine Lavazec

John Lok Man Law

Matthew B. Lawrenz

Benhur Lee

Jayhun Lee

Jean Claire Lee

Jiwon Lee

Patrick K. H. Lee

Seok-Yong Lee

Seung Jae Lee

Soo Chan Lee

Vincent T. Lee

Yong-Hwan Lee

Hsiang-Ting Lei

Salome Leibundgut-Landmann

Pushkar P. Lele

Jose A. Lemos

Jack C. Leo

Maria Lerm

Michele Leroux

Cammie F. Lesser

Michael Letko

Stuart M. Levitz

Gina R. Lewin

Kim Lewis

Zachary A. Lewis

Feng Li

Jianrong Li

Jie Li

Jonathan Z. Li

Min Li

Pengfei Li

Xianhe Li

Xin Li

Ya Li

Yen-Li Li

Yeqi Li

Yize Henry Li

Apostolos Liakopoulos

Chen Liang

Mary E. Lidstrom

Tami Lieberman

Bruno P. Lima

Andrew H. Limper

Jun Lin

Cecilia Lindestam Arlehamn

Richard James Lindsay

Roger G. Linington

Trevor Lithgow

Benjamin M. Liu

Chong Liu

Haoping Liu

Huiquan Liu

Hung-Jen Liu

Jiafeng Liu

Jinhua Liu

Pinghuang Liu

Qinfang Liu

Sanzhen Liu

Weida Liu

Wende Liu

Xiang Liu

Per O. Ljungdahl

Robert Lochhead

Frank E. Löffler

Catherine M. Logue

James K. Londino

Feng Long

Francisco Lonzano

Marcela de Freitas Lopes

Job E. Lopez

Juvenal Lopez

F. Xavier Lopez-Labrador

Richard Losick

Rogerio Ferreira Lourenco

Emma Kate Love

Andrew Lee Lovering

Susan T. Lovett

Jun Siong Low

Tiffany M. Lowe-Power

Ling Lu

Ti Lu

Xiaonan Lu

Jeremy Luban

Piotr Łukasik

Brian Michael Luna

Kenneth Lunstrom

Yi Luo

Zhao-Qing Luo

Carolina Luquez

Tal Luzzatto-Knaan

Bing Ma

Lay-Sun Ma

Li-Jun Ma

Zhe Ma

Jessica Maccaro

Alberto P. Macho

Jason Mackenzie

Erich R. Mackow

Ravi Madduri

Hiten Madhani

Marisa Madrid

Gkikas Magiorkinis

Robbie B. Mailliard

Natalia Malachowa

Gathsaurie Neelika Malavigne

Iris Maldener

Joao Filipe Inacio Mamede

Gideon Mamou

Pierre Mandin

Lara Manganaro

Sinalo Mani

Balaji Manicassamy

Daniel Santos Mansur

Mark Manzano

Meng Mao

Yuxin Mao

Erick B. Maosa

Katrina Mar

Emmanouil "Manolis" Maragkakis

Alessandro Marcello

Andrés E. Marcoleta

Joe Marcotrigiano

Anthony Maresso

Anthony W. Maresso

Willm Martens-Habbena

David Martinez

Luis R. Martinez

José Luis Martínez

Manuel Martínez-García

Clara Martinez-Perez

Bruno Martorelli Di Genova

Stefano Marzi

Ameya A Mashruwala

Kevin M. Mason

Eric Massé

Orietta Massidda

Cyrille Mathieu

Renata C. Matos

Frederick Matsen IV

Yasufumi Matsumura

Hiroshi Matsuo

Keith R. Matthews

Kerrie L. May

Christopher Mayack

Andreas Mayer

Marcel Mayer

Jere W. McBride

Shonna M. McBride

Charley McCarthy

Mark S. McClain

Megan Nicole McClean

Ryan McClure

Malcolm J. McConville

Beth A. McCormick

John K. McCormick

John McCullough

Sarah McDonald Esstman

James B. McKinlay

Meagan McMahon

George B. McManus

Ryan P. McNamara

Alexander J. Meeske

Sahar Melamed

Victoria A. Meliopoulos

Stephen B. Melville

Vineet D. Menachery

Sandra Yadira Mendiola

Niu Mengyao

Paola E. Mera

Andres Merits

Jordan P. Metcalf

William W Metcalf

Julie L. Meyer

Charlotte Michaux

Paul Michels

Tam Mignot

Laura A. Mike

Christopher J. Miller

Corrie Miller

Daniel P Miller

Scott R. Miller

Erez Mills

Ross Milton

Tohru Minamino

Nana Minkah

Satish Mishra

Dominique Missiakas

Aaron P. Mitchell

Angela Marie Mitchell

Maria Mittag

Valerie Mizrahi

Harry L. T. Mobley

Edward S. Mocarski

Florian Moeller

Axel Mogk

Joachim Mohn

Anne Moir

Nardus Mollentze

Mohammad Moniruzzaman

Cary A. Moody

D. Branch Moody

Hector M. Mora-Montes

Benjamin Morehouse

Silvia N. J. Moreno

Christopher Morgan

Yasu S. Morita

Hiromitsu Moriyama

Cécile Morlot

Joachim Morschhäuser

Bernard Moss

Vladimir L. Motin

Shakeel Mowlaboccus

Craig L. Moyer

Patrick Moynihan

Jianbing Mu

Ryan Sean Mueller

Hayley Muendlein

Monica Mugnier

Elke Mühlberger

Budhaditya Mukherjee

Asish Kumar Mukhopadhyay

Biswarup Mukhopadhyay

Debanjan Mukhopadhyay

Jayanta Mukhopadhyay

Suchetana Mukhopadhyay

Mandy Muller

Marcel Müller

Conrad W. Mullineaux

Matthew A. Mulvey

Joshua Munger

Carol A. Munro

Eric Muraille

Katsuhiko Murakami

Pablo R. Murcia

Georgi Muskhelishvili

Peter J. Myler

Hannu Myllykallio

Indira Mysorekar

Vasanthi Nachiappan

Carey Nadell

Thomas Naderer

Beiyan Nan

David Natanov

William Wiley Navarre

Miguel Navarro

Dipti D. Nayak

Wilfred Ndifon

Danny Nedialkova

Dhar Neeraj

Stuart J. Neil

Chris Netherton

Jeniel E. Nett

Hayley J. Newton

Olivier Neyrolles

Johan Neyts

James C. K. Ng

Wai-Leung Ng

Nguyen Thi Khanh Nhu

Scott Nichols

Anthony V. Nicola

Masahiro Niikura

Pablo Ivan Nikel

Leonardo Nimrichter

Estanislao Nistal-Villan

Xuemei Niu

Patrick Murigu Kamau Njage

Justin R. Nodwell

Nicholas Noinaj

Minou Nowrousian

Takuro Nunoura

Francisca Obiageri Nwaokorie

Bridget O'Banion

David H. O'Connor

Charlotte Odendall

Peter Oelschlaeger

Lisa Oestereich

James P. O'Gara

Ursula Oggenfuss

Marco Rinaldo Oggioni

Amanda G. Oglesby

Myung Whan Oh

Andrew J. Olive

Rita Oliveira

Antonio Oliver

Michal A. Olszewski

Teresa R. O'Meara

Anders Omsland

Andrew B. Onderdonk

Masayuki Onishi

Luis Humberto Orellana

Mona Orr

Kim Orth

Almudena Ortiz-Urquiza

Megan Orzalli

Takashi Osanai

Ernest Osburn

Nir Osherov

Kyla S. Ost

Karen M. Ottemann

Michael Otto

Daniel E. Otzen

Michelle A. Ozbun

Stefano Pagliara

Utpal Pal

Gustavo F. Palacios

Guy Palmer

Timothy Palzkill

Jianyi Pan

Yasi Pan

Amit Kumar Pandey

Gunjan Pandey

John C. Panepinto

Yi-Chun Pang

Xiaowu Pang

Ralph Panstruga

Kai Papenfort

Bernadett Papp

Coral Pardo-Esté

Alexander R. Paredez

Romain Parent

Laura Parfrey

Joanna L. Parish

Eunsook Park

Hee-Soo Park

Seoryeong Park

Yoonkyung Park

Dane Parker

John Everett Parkinson

Vaidehi Patel

Ranjana Pathania

Michael Patnode

Kathryn Patras

Kristin L. Patrick

Atmika Paudel

Ian T. Paulsen

William A. Paxton

Andrew Pekosz

Philip Pellet

Hernán F. Peñaloza

Miguel A. Penalva

Guiqing Peng

Xinxia Peng

Fatima Pereira

Daniel R. Perez

Christian Pérez

Daniel Pérez-Núñez

Juan Perilla

Stanley Perlman

Steve Perlman

Matthieu Perreau

Andreas Peschel

Christopher W. Peterson

William Petri

Eugen Pfeifer

Meg Philips

Anna Phillips

Mariana G. Pinho

Diana P. Pires

Johann Pitout

Javier Pizarro-Cerda

Vicente Planelles

Paul J. Planet

Roy Neal Platt

Alexander Ploss

Stefan H. Pöhlmann

Laurent Poirel

Albert Pol

Grace Pold

Angel Porgador

Owen Pornillos

Daniel A. Portnoy

Aimee Potter

Christopher Power

Rafael Prados-Rosales

Peter Preiser

Andrew Preston

Suzette A. Priola

Sladjana Prisic

Corinna Probst

Diana Proctor

Jonathan Pruneda

Dov Prusky

Carla Pruzzo

Michael Pucci

Erin B. Purcell

John G. Purdy

Aaron Webster Puri

Wei Qian

Chuanling Qiao

Udi Qimron

Xuebin Qin

Hua-Ji Qui

Lisa Racki

J. Pablo Radicella

Manuela Raffatellu

Jean-Baptiste Raina

Ricardo Rajsbaum

Katherine S. Ralston

Mika Rämet

Peter W. Ramirez

Irene Ramos

Christopher Rao

Vivek Rao

Xiancai Rao

Rubhana Raqib

Philip N. Rather

Alison Ravenscraft

John F. Rawls

Fernando Real

Johannes G. Rebelein

Shane Redelinghuys

Matthew Reeves

Gemma Reguera

Juan Reguera

Courtney Reichhardt

Alan Rein

Aaron Reinke

David Rekosh

Olaya Rendueles

Federico Rey

Felix A. Rey

Todd B. Reynolds

Johanna Rhodes

Michael H. J. Rhodin

Inna Ricardo-Lax

Louis Rice

Peter Rice

Virginia I. Rich

Anthony R. Richardson

Karen Ridge

Christian U. Riedel

Shannon Esher Righi

Sebastián Riquelme

Jeanine Rismondo

Juan Rivera-Correa

Tahir A. Rizvi

Dwayne Roach

Chris Robinson

Gene Robinson

Mélanie Roch

Eduardo P. C. Rocha

Daniel D. Rockey

Chathuraka Rodrigo

Marcio Rodrigues

Rodrigo A. L. Rodrigues

Holger Rohde

Ronda J. Rolfes

Morgane Rolland

Jeffrey A. Rollins

Nicolas Romero

James C. Romero-Masters

Jesus A. Romo

Severin Ronneau

R. Martin Roop

Maria Manuela Rosado

Federico Rosconi

Alex Rosenberg

Philip J. Rosenthal

Emily E. Rosowski

Brittany Ross

Ted M. Ross

Andrew Roth

Alissa Rothchild

Brice Rotureau

Thomas Rudel

Paulami Rudra

Natividad Ruiz

Till Rumenapf

Laura N. Rusche

Edward T. Ryan

Jeroen P. J. Saeij

Saveez Saffarian

Jason W. Sahl

Linda J. Saif

Kohta Saito

Suzana Pinto Salcedo

Maria-Carla Saleh

Padmini Salgame

Jon Salmanton-García

Guy Salvesen

John C. Samuelson

Johan K. Sandberg

Nicholas Sandoval

Christophe Sandt

Felipe H. Santiago-Tirado

Pierre Santucci

Kaustuv Sanyal

Martin Sapp

Jaakko Saraste

Christopher Sassetti

Karla J. F. Satchell

Sampurna Sattar

John-Demian Sauer

Karin Sauer

Ram Savan

Mohammad Savari

Sarah Scalercio

Charles A. Scanga

Dave J. Scanlan

Lennart Schada Von Borzyskowski

Birgit E. Scharf

Mark Schembri

Artur Scherf

Susan Schlimpert

Mirco Schmolke

John Schoggins

Robert T. Schooley

Michael Schotsaert

Tony Schountz

Jared M. Schrader

Sabrina Schreiner

Hinrich Schulenburg

Julia Schumacher

Michael J. Schurr

Ilan Schwartz

Julia Schwartzman

Jessica A. Scoffield

Kathleen M. Scott

Martha Sedegah

Markus Seeger

Gil Segal

Cecile Segonzac

Michael Seidl

Shlomo Sela (Saldinger)

Barbara Seliger

Adnane Sellam

Anna K. Serquiña

Seth Seth Bloom

William M. Shafer

Kathy Ho Yen Shair

Tongling Shan

Robert M. Q. Shanks

B. Jesse Shapiro

Pushkar Sharma

Amit Sharma

Dana Shaw

Lindsey N. Shaw

Qunxin She

Timothy P. Sheahan

Lilach Sheiner

Aimee Shen

Binglei Shen

Zeyang Shen

Kelly M. Shepardson

Yu Shi

Michael U. Shiloh

Masaki Shintani

Francesca L. Short

Naglaa H. Shoukry

Joshua D. Shrout

Deepak Shukla

Shantanu Shukla

Iart Luca Shytaj

Bupe A. Siame

Amam Zonaed Siddiki

Chandni Sidhu

M. Sloan Siegrist

Susanne Sievers

Paul Sigala

Mark W. Silby

Cecilia Alejandra Silva-Valenzuela

Jared A. Silverman

Anthony P. Sinai

John Sinclair

Varsha Singh

Amit Singhal

Barbara S. Sixt

Eric P. Skaar

Pamela J. Skinner

Jan Slapeta

Richard Sloan

Mark S. Smeltzer

Amber M. Smith

Evan S. Snitkin

Jonathan W. Snow

Ray T. Y. So

Gloria Soberon-Chavez

Liz Sockett

Beate Sodeik

Kenneth Söderhäll

Paul Soma

Hokyoung Son

Bronte Sone

Daniel E. Sonenshine

Jeongmin Song

Yang Song

Yongcheng Song

Erica Sonnenburg

Joseph A. Sorg

Victor Sourjik

Filipa L. Sousa

Gerald Spaeth

Brady Spencer

Tobias Spielmann

Shiranee Sriskandan

Anand Srivastava

Martin St. Maurice

Christopher Staley

Catherine Stanton

Kenneth A. Stapleford

Michael Starnbach

Tyler Starr

Chad Steele

Ida Helene Steen

Jacob Steenwyk

Benjamin Stein

Madison Elaine Stellfox

Yvon Sterkers

Brian Stevenson

Scott Stibitz

Boris Striepen

Birgit Strommenger

Frank Stubenrauch

Bin Su

Agathe Subtil

Gurol Suel

Sho Sugawara

Andreas Suhrbier

Christopher Sullivan

David Sullivan

William Sullivan

Guorong Sun

Haipeng Sun

Liying Sun

Xiao Sun

Xumei Sun

Yan Sun

Matthew Surette

Bas Surewaard

Susmit Suvas

Elena Suvorova

Nobuhiro Suzuki

Chiho Suzuki-Minakuchi

Staffan Svard

Chad Swanson

Christine Szymanski

Jan Tachezy

Michiko Taga

Constantin Takacs

Norio Takeshita

David Talmy

Rita Tamayo

Gene Tan

Ming Tan

Shanjun Tan

Shumin Tan

Biao Tang

Christoph Tang

Martin Taubert

Amalio Telenti

Sam Telford

Italo Tempera

Marcus Teixeira

Michelle Tate

Olivier Terrier

Rita Tewari

Tinkara Tinta

Anna Tischler

Alfredo Torres

Nathanial Torres

Micaela Tosi

Frances Trail

Ana Traven

Cagla Tukel

Massimo Turina

David Turra

Kevin Tyler

Osvaldo Ulloa

Taylor Updegrove

Lucas Ustick

Daniela Valone

Raphael Valviddiva

Jolien Van Cleemput

Bert van den Berg

Adrianus W. M. van der Velden

Brian C. VanderVen

Tom Van de Wiele

Janneke H. H. M. van de Wijgert

Giel G. van Dooren

Gregory Van Duyne

Rachel Van Duyne

Linda F. van Dyk

Mark W. J. van Passel

Norman van Rhijn

Peter van Ulsen

Jose Vargas-Muniz

Tommi Vatanen

Thomas Ve

Anthony Vecchiarelli

Daniel E. Vélez-Ramírez

Vittorio Venturi

Abhishek Verma

Laura Veschetti

Laura Villanueva

Nikolaos Vlachogiannis

Anastasia N. Vlasova

Joseph Vogel

Thomas Voss

Jatin Vyas

Samuel Wagner

Shayne Yin

Sunnie Yoh

Anna York

Peter Young

Tao Yu

Hasan Zaki

Ivan Zanoni

Merve Zeden

Bo Zhao

Xuhui Zheng

Jie Zhong

Oren Zimhony

Shenshen Zou

Kateryna Zhalnina

Zhi-Ming Zheng

Guohua Zhong

Wei Zhong

Jin Zhou

Shungui Zhou

Xi Zhou

Jieqing Zhu

Jingen Zhu

Jun Zhu

Yongqun Zhu

Yongzhang Zhu

Zixiang Zhu

Tomaž Mark Zorec

Zhengzhong Zou

Wolfram Zückert

